# Barriers to and Facilitators of Engaging With and Adhering to Guided Internet-Based Interventions for Depression Prevention and Reduction of Pain-Related Disability in Green Professions: Mixed Methods Study

**DOI:** 10.2196/39122

**Published:** 2022-11-09

**Authors:** Lina Braun, Johanna Freund, Janika Thielecke, Harald Baumeister, David Daniel Ebert, Ingrid Titzler

**Affiliations:** 1 Department of Clinical Psychology and Psychotherapy Institute of Psychology and Education University of Ulm Ulm Germany; 2 Department of Clinical Psychology and Psychotherapy Institute of Psychology Friedrich-Alexander-Universität Erlangen-Nürnberg Erlangen Germany; 3 Department of Sport and Health Sciences Professorship for Psychology and Digital Mental Health Care Technical University Munich Munich Germany

**Keywords:** internet-based intervention, depression, chronic pain, barriers and facilitators, qualitative research, uptake, adherence, farmers, gardeners, foresters

## Abstract

**Background:**

Internet-based interventions (IBIs) are effective for the prevention and treatment of mental disorders and are valuable additions for improving routine care. However, the uptake of and adherence to IBIs are often limited. To increase the actual use of IBIs, it is important to identify factors for engaging with and adhering to IBIs.

**Objective:**

We qualitatively evaluated barriers and facilitators regarding a portfolio of guided IBIs in green professions (farmers, gardeners, and foresters).

**Methods:**

Interview participants were selected from 2 randomized controlled trials for either the prevention of depression (Prevention of Depression in Agriculturists [PROD-A]) or the reduction of pain interference (Preventive Acceptance and Commitment Therapy for Chronic Pain in Agriculturists [PACT-A]) in green professions. The intervention group in PROD-A (N=180) participated in an IBI program, receiving access to 1 of 6 symptom-tailored IBIs. The intervention group in PACT-A (N=44) received access to an IBI for chronic pain. Overall, 41 semistructured qualitative interviews were conducted and transcribed verbatim. Barriers and facilitators were identified via inductive qualitative content analysis, with 2 independent coders reaching almost perfect intercoder reliability (Cohen κ=0.92). A quantitative follow-up survey (30/41, 73%) was conducted to validate the results. Subgroup analyses were performed based on intervention characteristics.

**Results:**

We identified 42 barriers and 26 facilitators, which we assigned to 4 superordinate categories related to the intervention (20 barriers; 17 facilitators), work (4 barriers; 1 facilitator), individual (13 barriers; 8 facilitators), and technical (5 barriers; 0 facilitators) aspects. Key barriers (identified by at least 50% of the interviewees) were *time-consuming work life* (29/40, 73%) and *time-consuming private life* (23/40, 58%). Similarly, the most frequently identified facilitators included *presence of* *motivation, curiosity, interest and perseverance* (30/40, 75%), *flexible time management at work* (25/40, 63%), and *support from family and friends* (20/40, 50%). Although agreement with barriers in the quantitative follow-up survey was rather low (mean 24%, SD 11%), agreement with facilitators was substantially higher (mean 80%, SD 13%). Differences in agreement rates were found particularly between intervention completers and noncompleters. Completers agreed significantly more often that perceived IBI success; being motivated, curious, interested, and perseverant; and having a persisting level of psychological strain have been facilitating. Noncompleters agreed more often with experiencing the e-coach contact as insufficient and technical problems as hindering for intervention completion.

**Conclusions:**

Based on these results, strategies such as customization of modules for more flexible and adaptive use; video chat options with the e-coach; options to facilitate social support by family, friends, or other participants; or using prompts to facilitate training completion can be derived. These approaches could be evaluated in further quantitative research designs in terms of their potential to enhance intervention use in this occupational group.

**Trial Registration:**

German Clinical Trials Register DRKS00014000, https://tinyurl.com/3bukfr48; German Clinical Trials Register DRKS0001461, https://tinyurl.com/ebsn4sns

## Introduction

### Background

The effectiveness of internet-based interventions (IBIs) is well established for depression treatment [1,2] and prevention [3]. IBIs can also be applied to effectively reduce disease-related disability in chronic somatic conditions such as chronic pain [4-7]. Guided IBIs for the treatment of mental and somatic disorders based on cognitive behavioral therapy have even been shown to be equally effective as face-to-face therapy [8].

However, low treatment adherence can be a limiting factor for treatment effectiveness in IBIs [9,10]. In quantitative studies, various factors were found to be possible predictors of intervention adherence, including guidance [11], the use of persuasive design elements [12,13], or individual factors such as planning [14]. Thus, much research in recent years has focused on optimizing IBIs to facilitate intervention adherence in participants [11,15-17], and barriers to IBI use might vary depending on different intervention aspects such as guidance level, focus on specific symptoms, or type of intervention.

Furthermore, participant characteristics such as female sex [18,19] and higher age [10,18] were identified as potential predictors of higher intervention adherence, whereas results for other characteristics such as education level are inconsistent [18,19]. As intervention adherence seems to vary systematically with some participant characteristics, this might indicate different requirements for the intervention in accordance with target groups. Thus, identifying these specific requirements to address them in IBIs could be a promising approach.

Although qualitative insights into the relevant factors for the use of and adherence to IBIs are scarce for specific treatment indications such as reduction of pain interference in chronic pain or addressing specific health problems (eg, insomnia, anxiety, and diabetes) as a risk factor for the development of depression, some insights already exist regarding IBIs aimed at the prevention or treatment of depression. Qualitative studies on the use of IBIs for prevention of depression in the workplace, treatment of depression comorbid with cardiovascular risk factors, or stress reduction in the workplace with different levels of guidance, each identified on a personal level, barriers such as lack of time, high stress levels or competing priorities, and low motivation because of negative mood or anxiety [20-22]. At a program level, barriers regarding content complexity and redundancy, program functionality, and perceived dangers such as privacy of the IBI were mentioned in a depression-prevention context targeting workers who were at high risk for depression [22]. Furthermore, aspects such as lack of personalization, lack of perceiving the IBI as therapy, or lack of new learnings because of known content were described with regard to an unguided IBI for depression treatment without therapeutic support [21]. Therefore, there is still a research gap to bridge regarding the identification of barriers to the use of a portfolio of guided IBIs to specifically address different health complaints as risk factors for the development of depression or pain-associated disability.

Barriers to and facilitators for the use of IBIs might even vary depending on the population being targeted. In rural contexts, specifically, barriers to mental health seeking have been reported to be higher than barriers to physical health seeking [23]. Furthermore, stigma against depression and lower agreement about depression treatment have been shown to be more prominent in rural than in urban contexts [24]. At the same time, the use of IBIs might be more acceptable to rural than to urban populations, as some studies have reported that rural populations have a lower preference for face-to-face contact than urban populations and are especially appreciative of autonomy and confidentiality aspects of IBIs, as indicated by a systematic review [25].

In the rural context, farmers seem to be especially at risk for mental disorders such as depression because of diverse risk factors such as financial strain, dependency on weather conditions, government regulations, high work demands, or psychosocial difficulties [26-30]. Furthermore, the prevalence of musculoskeletal pain symptomology is higher in farmers than in nonfarmers because of physical strain in agricultural activities [31,32]. Thus, pain interference with work and everyday activities can be assumed to be an additional burden in this occupational group. Thus, a research gap exists in identifying barriers to and facilitators of IBI use in the occupational group of farmers who are at risk for depression or are burdened with chronic pain.

Guided IBIs have been investigated in the specific occupational group of green professions, including farmers, gardeners, and foresters, as part of the model project “With us in balance,” initiated by a social insurance in Germany regarding their effectiveness in reducing depressive symptomology (Prevention of Depression in Agriculturists [PROD-A]; trial registration: DRKS00014000) and pain interference (Preventive Acceptance and Commitment Therapy for Chronic Pain in Agriculturists [PACT-A]; trial registration: DRKS00014619) in 2 separate randomized controlled trials (RCTs) [33,34]. The first effectiveness results of a tailored IBI program aimed at the prevention of depression by targeting various risk factors revealed low intervention adherence in this target group, with only 22.2% of the intervention group completing at least 80% of the intervention modules 9 weeks after randomization [35], 51.5% at 6-month and 55.6% of the intervention group at 12-month follow-up [36]. These results are low in comparison with an average completer rate of 67.5% in guided IBIs for depression treatment [37] as well as a completer rate of 74.3% in an RCT evaluating a guided IBI for depression prevention in adults with subthreshold depression, each for the completion of at least 80% of the respective IBI [38]. This indicates challenges in the use of IBIs in this occupational target group. Therefore, determinants of uptake and adherence in this specific target group need to be investigated to successfully implement IBIs as part of routine health care [39].

### Objectives

To the best of our knowledge, there has been no research regarding barriers to and facilitators for the use of IBIs in the specific target group of green professions. In a first step of this mixed methods study, we aimed to uncover barriers to and facilitators for the uptake of and adherence to IBIs based on a qualitative content analysis of semistructured qualitative interviews conducted in this specific occupational group. In a second step, we contrasted agreement rates to the identified barriers and facilitators collected in a follow-up questionnaire with the number of mentions based on the qualitative interviews to validate the factors identified in the interview sample. In a third step, we exploratively investigated differences in agreement rates to the identified barriers and facilitators between groups with different treatment indications and in intervention completers versus noncompleters.

## Methods

### Study Setting and Design of the RCTs

Semistructured qualitative interviews were conducted as part of a mixed methods evaluation in the context of the 2 RCTs, PROD-A [33] and PACT-A [34]. Both RCTs are part of a preventive model project of the social health care insurance for farmers, gardeners, and foresters (Sozialversicherung für Landwirtschaft, Forsten und Gartenbau) in Germany called “*With us in balance*” and thus evaluate both the entire portfolio of IBIs provided to the target group of green professions. Both studies aimed to evaluate the clinical and cost-effectiveness of guided IBIs in green professions compared with enhanced treatment as usual. PROD-A evaluates a program of 6 IBIs for indicated prevention of depression in participants with at least subthreshold depression, whereas PACT-A evaluates an IBI for the reduction of pain-related disability in participants with chronic pain symptomology for a duration of at least 6 months. Participation was accessible to entrepreneurs, collaborating spouses, family members, and pensioners working in green professions aged ≥18 years with sufficient insurance status. Recruitment for both RCTs started in January 2018 using a combined recruitment strategy based on a joint web-based screening and was completed for PROD-A (N=360) in April 2019; for PACT-A, recruitment was prematurely terminated in July 2020 because of overall low recruitment success (N=89 instead of the planned N=256). Further details on the RCTs can be found in the corresponding study protocols [33,34].

### Ethics Approval

Both trials were approved by the Ethics Committee of the University of Ulm and registered in the German Clinical Trials Registry (DRKS00014000 and DRKS0001461). Informed consent was provided by all participants in both RCTs.

### The IBIs

PROD-A evaluated a tailored IBI program consisting of 6 different IBIs aimed at the prevention of depression or at risk factors for depression. The trainings were provided by an external service company (GET.ON Institute). The IBI program included the training *GET.ON Mood Enhancer*, aiming at depressive symptoms in general [40], as well as *GET.ON Mood Enhancer Diabetes* specifically for patients with comorbid diabetes [41]. Further trainings were *GET.ON Stress* focusing on issues with perceived stress [42], *GET.ON Recovery* for insomnia [43], *GET.ON Panic* focusing on panic and agoraphobic symptoms [44], and *GET.ON Be clever—drink less* thematizing problematic alcohol consumption [45]. PACT-A evaluated the training *GET.ON Chronic Pain* that focused on chronic pain symptomology [46] and aimed to improve pain-related disability based on acceptance and commitment therapy.

Participants in both intervention groups went through the following 3-step process: (1) participating in a psychodiagnostic web-based assessment to determine relevant symptom areas and risk factors, (2) having an initial contact with their assigned personal e-coach (trained and qualified psychologists, psychologists in training for psychotherapy, or trained psychotherapists) via telephone or internal messaging function, and (3) starting the training phase in the assigned IBI. For PROD-A participants, the initial contact was used for a shared decision-making process to choose the most suitable IBI, whereas PACT-A participants were directly assigned to GET.ON Chronic Pain.

All 7 IBIs contained 6 to 8 modules, with the recommendation to complete 1 module per week. The IBIs were guided by e-coaches, who gave feedback to participants on each completed module either via telephone or in written form on the intervention platform. The *training phase* was followed by a *consolidating phase*, in which participants could have short monthly contact with their e-coach for up to 12 months. IBIs were customized by the external service provider to the occupational group of green professions by adapting personas and examples to the agricultural context and including corresponding photo material. Further intervention details can be found in the corresponding study protocols of PROD-A [33] and PACT-A [34].

### Design of the Mixed Methods Study

A qualitative interview study was conducted with participants of the respective intervention arms of both RCTs, each of whom used 1 of the 7 guided IBIs. Recruitment and data collection of interview participants were either conducted by the study team of the Friedrich-Alexander-Universität (for PROD-A) or by the study team of the University of Ulm (for PACT-A). Additional informed consent was obtained from all interview participants. Semistructured interviews were conducted based on an interview guide addressing perceived barriers to and facilitators for the uptake of and adherence to the IBIs. The same interview guide was used for all participants, as the interview items were applicable to participants of both RCTs, and the qualitative data analysis was aimed at addressing the pooled transcripts from both RCTs. The interview questions were embedded in a broader interview guide addressing different topics pertaining to the use of the IBIs beforehand. The results are reported according to the COREQ (Consolidated Criteria for Reporting Qualitative Research) guidelines [47], as detailed in Multimedia Appendix 1. A quantitative follow-up survey was conducted to validate the results of the qualitative interview study. Interview participants were invited to report whether they agreed with the identified barriers and facilitators. Finally, statistical comparisons between specific subgroups (ie, treatment indication and completer status) were made based on the quantitative agreement rates.

### Recruitment and Data Collection Procedure

Recruitment for the qualitative interviews started in June 2019 (PACT-A) and August 2019 (PROD-A), as enrollment for both RCTs was either already completed or nearly completed (intervention groups—PROD-A: N=180; PACT-A: n=42 of overall n=44). Participants who had previously agreed to be contacted for further studies (PROD-A: n=161; PACT-A: n=39) received a standardized invitation letter via email. The recommended period for the completion of the assigned IBI had expired for all invited RCT participants at this point. Informed consent was received from 17% (27/161) of PROD-A participants and 49% (19/39) of PACT-A participants, and an appointment for the qualitative interview was scheduled.

Interview conduct was based on purposeful theoretical sampling [48], aiming to recruit an interview sample with maximum variation regarding participant characteristics, particularly sex, occupational role, completer status, and type of IBI received. Participants were defined as intervention completers if they had completed all available intervention modules in their respective IBI until the interview was conducted. Participants not reaching this criterion were categorized as noncompleters. The interviews were concluded after 41 interviews (PROD-A: N=22, PACT-A: N=19).

Interviews were conducted via telephone by 3 master’s degree candidates (Manuela Gasde, Andrea Riedel, and Saskia Locker) based on an interview guide and supervised by researchers Johanna Freund and Lina Braun. Interviews were audio recorded and transcribed verbatim based on an extended manual detailing transcription rules [49]. Personal details were anonymized [50] and participants were referenced with their study ID numbers. PROD-A participants did not receive compensation, whereas PACT-A participants received an expense allowance of €20 (US $22) for participating in the interview. The interview participants were invited to a quantitative follow-up survey for the validation of the identified themes. Participation in the follow-up survey was not compensated.

### Interview Guide

The interview items for answering the research question regarding relevant barriers and facilitators for the use of and adherence to IBIs were formulated by following the interview guide of a qualitative interview study conducted in a different application context [51]. The interview items were adapted to the context of IBIs and target groups and formulated using an inductive exploratory approach, aiming to generate broad and unconstrained information about possible barriers and facilitators for the use of IBIs in the specific target group of green professions. The chosen inductive exploratory approach was the most suitable one, as there is, to our knowledge, no theoretical framework describing barriers to and facilitators for the use of a tailored IBI portfolio in such a specific occupational target group. Furthermore, this approach allowed us to address different aspects regarding the broad portfolio of IBIs against the occupational context of green professions. The interview guide contained instructions for interview conduct and formulated 5 main items, each with subordinate items entailing prompts for specific aspects (called “memos”), follow-up questions for further elaboration (called “hang-on”) as well as filter questions to guide and standardize interview conduct for specific cases. The interview guide is featured in Table 1.

**Table 1:** Interview items for evaluating barriers and facilitators for the use of and adherence to the internet-based interventions.

Item number	Interview item
1^a^	How would you rate the training offer overall? What do you think of the offer?
1.1^b^	To what extent was the internet-based training suitable for you and your needs?
1.2^b^	To what extent were there aspects of the internet-based training that were not suitable for you and your needs?
2^a^	What helped you to “stick with” the internet-based training?
2.1^c^	Were there certain aspects that made it easier for you to continuously participate in the internet-based training?
2.2^b^	What personal circumstances made it easier for you to participate in the internet-based training?
2.3^b^	What professional circumstances made it easier for you to participate in the internet-based training?
3^a,d^	If you have ever been unable to engage with the internet-based training: what prevented you from engaging with the training and its content?
3^a,e^	You dropped out of the internet-based training after lesson (add number). What prevented you from engaging with the training and its content?
3.1^c^	What else prevented you from engaging with the content of the internet-based training?
3.2^b^	Specifically, was there anything that bothered you about the guided internet-based training that made you not want to engage with it?
3.3^b^	What personal circumstances prevented you from participating in the internet-based training?
3.4^b^	What professional circumstances prevented you from participating in the internet-based training?
3.5^b,e^	You have just described various problems. What was the decisive reason that you dropped out of the training?
4^a^	You just described that in (paraphrase situation) it was difficult for you to engage with the internet-based training. What would have helped you engage with the internet-based training in that situations?
4.1^c^	What else helped you?
4.2^b^	What else could have helped you in the internet-based training itself?
4.3^b^	Was there anything in your personal environment that could have helped you or someone who could have supported you?
4.4^b^	What role do friends and family play in supporting you to participate in the training?
4.5^b^	What positive reactions do you remember?
4.6^b^	What negative reactions were there?
4.7^b^	Was there anything in your professional context that could have helped you or someone who could have supported you?
5^a^	Imagine you would participate in an internet-based training again. Imagine also that you could wish for an internet-based training that would be exactly suitable for your needs. What would the ideal internet-based training look like for you?
5.1^c^	How would the internet-based training need to be designed to make you feel that you are basically capable of doing the internet-based training regularly and stick with it until completion?
5.2^c^	How would your private and professional environment have to be organized to make you feel that you are basically capable of doing the internet-based training regularly and stick with it until completion?

^a^Main question.

^b^Memo.

^c^Hang-on.

^d^Filter question for completers.

^e^Filter question for noncompleters.

### Data Analysis

#### Qualitative Analysis

Qualitative content analysis was conducted based on 40 verbatim interview transcripts from both RCTs, using inductive category development [52]. One transcript had been excluded beforehand because it could not be objectively verified based on data from the intervention platform that the IBI was actually started by the participant and the reliability of his statements was doubtful. The codes were derived from the raw material with regard to the research question, using the procedure described as follows:

1. The 40 transcripts were divided into 10 equal portions of material, consisting of 4 interviews in each portion. The selection of the transcripts was balanced for participant characteristics, predominantly for sex, occupational role, completer status, and treatment indication (ie, PROD-A and PACT-A).
2. The first 10% of the material was independently inspected by 2 coders (Sophie Pausch and Lea Beywl) who generated codes from the material based on the research question. The generated codes were discussed until both coders agreed on a preliminary code system. This preliminary code system was then complemented by code definitions and exemplary statements from the interviews. Then, the current code system was discussed and reflected upon in a consensus meeting between the coders and the supervisor (Lina Braun) to resolve questions, reach a consensus on differing viewpoints, and ensure that the coding was done in accordance with the research question. Subsequently, the code system was adapted by Sophie Pausch.
3. This iterative procedure was then repeated by including the next portion of 4 interviews in the raw material. Both coders independently reviewed and modified the preliminary code system based on the new material, taking into account the already coded material. The coders discussed their adaptations and agreed on a preliminary modified code system. This version of the code system was discussed once again in a consensus meeting with the supervisor Lina Braun and was modified according to the consensus reached. The iterative coding procedure was then continuously repeated by extending the coding material to the next 4 interviews in each coding pass.
4. After the seventh iteration (ie, after 28 of the 40 interviews were included in the coded material), the coding system was additionally reviewed in detail by Lina Braun to ensure a distinct code allocation and a differentiated abstraction level of the code system. Sophie Pausch included this feedback in the revised code system.
5. After the eighth coding pass with an additional 10% portion of interviews, with overall 32 interview transcripts being included in the iterative development of the code system, it was concluded that theoretical saturation had been reached, as no inherently new category was added.
6. Coding rules were parallel to the development of the code system (steps 2 to 5) continuously developed, discussed, and finalized in the consensus meetings.
7. On the basis of the finalized code system and coding rules, the complete material, that is, the 40 interview transcripts, were independently coded by 2 coders (Sophie Pausch and Lea Beywl) in 1 pass. As no necessity for further consensus meetings arose during the coding process, coder independence during the final coding process was maintained. The intercoder reliability was exceptionally high (Cohen κ=0.92) and can be classified as almost perfect [53] based on the Brennan-Prediger coefficient κ [54].
8. To ensure communicative validity [55] of the identified themes, we presented them to the interviewed participants after the completion of the data analysis in a web-based follow-up survey. The themes were presented based on definitions but without quotations, and participants were instructed to rate whether they agreed with the hindering and facilitating factors described.

Verbatim transcription and qualitative data analyses were conducted using the data analysis tool MAXQDA (version 2018.2; VERBI Software GmbH) [56].

#### Quantitative Analysis

For sociodemographic comparisons, 2-tailed *t* tests were conducted for continuous variables and Fisher exact tests were conducted for categorical variables. Furthermore, explorative subgroup analyses for completer status and treatment indication were conducted based on dummy-coded quantitative variables from the quantitative follow-up survey. Subgroup analysis based on Fisher exact test was conducted for each barrier and facilitator based on the frequency of agreement to each factor for each group in the quantitative follow-up survey. Fisher exact test was chosen because of its robustness in small sample sizes and its cell counts often being <5 [57]. For all analyses, 2-sided *P* values were reported with *P*<.05 being used for assuming statistical significance. Quantitative analyses were conducted using SPSS Statistics (version 26; IBM Corp) [58].

## Results

### Participant Characteristics

Participant characteristics were analyzed for the total interview sample of 41 interviewees as well as subgroups of completers and noncompleters, as detailed in Table 2. Intervention use was analyzed with a focus on representativeness in relation to the main trials, which is why the interview sample was assembled to include at least one participant per IBI. The intervention use of the interview participants was in line with the overall intervention use in the PROD-A RCT, with GET.ON Stress being the IBI assigned most often along with GET.ON Mood Enhancer and GET.ON Recovery [35,36]. The ratio of interview participants who completed all intervention modules (completers) until the time of the interview to participants who did not complete all intervention modules (noncompleters) was 3:2. By contrast, the ratio of completers to noncompleters until the 12-month follow-up in the main trials (ie, PROD-A and PACT-A) was approximately 1:1. Multimedia Appendix 2 displays participant characteristics of the interview subsamples of PROD-A and PACT-A along with the total intervention samples of both RCTs to show the degree of representativeness of the interview sample for the main studies.

**Table 2:** Comparison of the characteristics of completers and noncompleters of the interview sample (N=41).

			All interview participants		Completers^a^ (n=24)		Noncompleters^b^ (n=17)		*t* test (*df*)^c^		*P* value^d^	
**Sociodemographic characteristics**												
	Sex (male), n (%)	17 (41)		8 (33)		9 (53)		N/A^e^		.34		
	Age (years), mean (SD)	55.88 (7.86)		54.71 (8.36)		57.53 (6.99)		1.14 (39)		.26		
	In a partnership or married, n (%)	39 (95)		23 (96)		16 (94)		N/A		>.99		
**Education, n (%)**												.67
	Low	18 (44)		10 (42)		8 (47)		N/A				
	Middle	13 (32)		9 (38)		4 (24)		N/A				
	High	10 (24)		5 (21)		5 (29)		N/A				
**Occupational role, n (%)**												.91
	Entrepreneur	16 (39)		9 (38)		7 (41)		N/A				
	Contributing spouse	12 (29)		7 (29)		5 (29)		N/A				
	Contributing family member	5 (12)		3 (13)		2 (12)		N/A				
	Pensioner or spouse of pensioner	6 (15)		3 (13)		3 (18)		N/A				
	Incapacitated for work	2 (5)		2 (8)		0 (0)		N/A				
**Study affiliation, n (%)**												.54
	PROD-A^f^	22 (54)		14 (58)		8 (47)		N/A				
	PACT-A^g^	19 (46)		10 (42)		9 (53)		N/A				
**Type of internet-based intervention, n (%)**												.58
	GET.ON Mood Enhancer	5 (12)		2 (8)		3 (18)		N/A				
	GET.ON Stress	11 (27)		7 (29)		4 (24)		N/A				
	GET.ON Recovery	2 (5)		2 (8)		0 (0)		N/A				
	GET.ON Panic	1 (2)		1 (4)		0 (0)		N/A				
	GET.ON Be Clever—Drink Less	2 (5)		2 (8)		0 (0)		N/A				
	GET.ON Mood Enhancer Diabetes	1 (2)		0 (0)		1 (6)		N/A				
	GET.ON Chronic Pain	19 (46)		10 (42)		9 (53)		N/A				
**Period between baseline and interview (months), n (%)**												.11
	<6	2 (5)		0 (0)		2 (12)		N/A				
	6-12	14 (34)		7 (29)		7 (41)		N/A				
	>12	25 (61)		17 (71)		8 (47)		N/A				

^a^Completers were defined as interview participants who completed all intervention modules until the time of the interview.

^b^Noncompleters were defined as interview participants who had not completed all intervention modules until the time of the interview.

^c^*t* test was used only for continuous variables.

^d^*P* value is based for continuous variables on a 2-tailed *t* test and for categorical variables on an exact Fisher test.

^e^N/A: not applicable.

^f^PROD-A: Prevention of Depression in Agriculturists.

^g^PACT-A: Preventive Acceptance and Commitment Therapy for Chronic Pain in Agriculturists.

### Qualitative Findings

Qualitative analysis identified 42 barriers and 26 facilitators that were categorized into the following main categories: (1) *intervention-related factors,* with the subcategories *training content* (barriers: 7/42, 17%; facilitators: 6/26, 23%) and *training realization and design* (barriers: 13/42, 31%; facilitators: 11/26, 42%); (2) *work-related factors* (barriers: 4/42, 10%; facilitators: 1/26, 4%); (3) *individual-related factors* (barriers: 13/42, 31%; facilitators: 8/26, 31%); and (4) *technical-related factors* (barriers: 5/42, 12%; facilitators: 0/26, 0%)*.*

Overall, 2 barriers (1) *time-consuming work life* (29/40, 73% of interviewees) and (2) *time-consuming private life* (23/40, 58%) as well as 3 facilitators, *flexible time management at work* (25/40, 63%); *presence of motivation, curiosity, interest, and perseverance* (30/40, 75%); and *support from family and friends* (20/40, 50%), were mentioned by at least 50% (20/40) of the interviewed sample and thus identified as key themes. Most of the identified factors represent a broad range of different aspects that can hinder or improve the participation in an IBI. Furthermore, 55% (22/40) of barriers and 62% (16/26) of facilitators were mentioned by at least 10% (4/40) of the interviewees and thus are listed as major themes with definitions and exemplary statements in Tables 3-6. The remaining barriers (20/42, 48%) and facilitators (10/26, 38%) that were reported by <4 interviewees (<10%) are summarized in Multimedia Appendix 3 as we assumed these factors to be of less relevance to the target group. Of these, 15% (6/40) of barriers and 27% (7/26) of facilitators were addressed by only 1 interviewee each.

On an average, interviewees named 6 barriers (SD 2.8; range 2-14) and 5 facilitators (SD 2.7; range 1-10). Intervention completers (n=24) named on average 6 barriers (SD 2.9; range 2-14) compared with noncompleters (n=16) reporting on average 7 barriers (SD 2.5; range 2-12). This difference was not statistically significant (*t*_38_=1.1; *P*=.27). Furthermore, intervention completers named on average 6 facilitators (SD 2.5; range 2-10), compared with noncompleters reporting on average 4 facilitators (SD 2.5; range 1-9). This difference was statistically significant (*t*_38_=−2.4; *P*=.02).

**Table 3:** Major themes of the qualitative results for the intervention-related barriers and facilitators pertaining to internet-based intervention (IBI) content from participants’ perspectives (mentioned by at least 4 participants; N=40).

Categories		Participants			Definition		Supporting quotations
		Values, n (%)	Number of excerpts^a^				
**Intervention-related barriers, IBI content (n=5)^b^**							
	Unhelpful content	11 (28)	18	Participants perceived the IBI content as unhelpful, uninformative, and uninteresting.		“Well, there was one or the other exercise that...I liked less or where I had less interest in it...when there are several exercises, that there is always a favorite and there is one that you don’t like so much.” [Interview 26]	
	Impersonal or static content	10 (25)	23	The content of the IBI (eg, specific exercises and questions) was perceived to be static, as in not being tailored to the participant, not addressing personal problems not, or not providing the option to select or deselect topics.		“It’s not very personal, it’s a machine.” [Interview 5]	
	Missing key topics or unappealing focus	5 (13)	11	Participants mentioned that key topics were missing (eg, IBI content on dealing with aging in the green professions and IBI content with movement and sports exercises) or that they found the focus of the IBI (eg, on psychological support) to be unappealing.		“For my needs, I’m telling you, I was concerned with chronic pain, not psychological support.” [Interview 4]	
	Level of requirements being perceived as too high or low	5 (13)	8	The level of requirement was perceived as too low (eg, if IBI contents were already known before the start of the IBI) or too high (eg, if the person was severely ill).		“...I think it fit for mild cases who only feel overwhelmed now and then.... Whether it is fitting for someone who is on the verge of burnout, I dare to doubt that.” [Interview 30]	
	Difficulty in identifying with exemplary personas	4 (10)	4	Participants reported difficulties in identifying with the exemplary personas described in the IBI modules.		“...In the first two lessons, there was always a reference to these people that you introduced. One of them was pushed by a bull.... Well, I don't know if I could be pulled down like that by such an accident....” [Interview 4]	
**Intervention-related facilitators, IBI content (n=4)^c^**							
	Helpful content	14 (35)	20	Participants perceived the content to be helpful, informative and interesting.		“I found, the information about the disease VERY helpful or, well. I found it informative and educational!” [Interview 29]	
	Engagement with one’s problems	12 (30)	15	Participants found it helpful to reflect on themselves and their problems, to become aware of their problems, and to do something good for themselves.		“It just did me good to deal with myself again. That I, um...consciously do something for myself.” [Interview 38]	
	E-coach support	6 (15)	9	Participants perceived the personal contact, the exchanges with the e-coach and the feedback from the e-coach as helpful.		“The contact with the e-coach helped. That you always get feedback, questions and...notice that the [incomprehensible] are appreciated, which you have done....” [Interview 34]	
	Perceiving the IBI as a further health care approach	5 (13)	7	Participants perceived the IBI as a new, eventually promising treatment option.		“I think it’s a super great thing because you just don’t get anywhere with other things and it’s a good way to A) deal with the issue and B) deal with broader issues too.” [Interview 13]	

^a^Total number of excerpts, including multiple mentions from the same persons.

^b^Factors related to the IBI content (eg, specific exercises), that made it difficult to participate in the IBI.

^c^Factors related to the IBI content (eg, specific exercises), that made it easier to participate in the IBI.

**Table 4:** Major themes of the qualitative results for the intervention-related barriers and facilitators pertaining to internet-based intervention (IBI) realization and design from participants’ perspectives (mentioned by at least 4 participants; N=40).

Categories		Participants			Definition		Supporting quotations
		Values, n (%)	Number of excerpts^a^				
**Intervention-related barriers, IBI realization and design (n=6)^b^**							
	IBI modules being too long	16 (40)	28	The IBI modules and the time needed to complete them were perceived as too long, and shorter and more frequent IBI modules would have been preferred.		“Only the length was too much at once. It’s better to have short lessons more often than to sit at them for an hour and even longer or two hours each time. That’s too much in terms of length, yes,...in terms of required time.” [Interview 11]	
	Limited or complicated access to IBI content	10 (25)	16	Participants perceived the access to the contents of the IBI as limited or complicated and reported not having been able to access specific contents directly (eg, access to already completed exercises, questions, and audio files).		“...which was a bit inconvenient that you then always had to go to the next page within a lesson. Especially in the post-processing, where I knew there was this one point. But I had to look through all these pages before I got to that point....” [Interview 23]	
	Insufficient personal e-coach contact	10 (25)	15	The e-coach contact was perceived as not personal enough because of little use of the feedback option via telephone or the lack of face-to-face conversations and the resulting anonymity. Participants expressed the wish for more personal conversations, explanations regarding the IBI, and overall more telephone contact.		“Maybe this, what do you call it, this anonymity.... Well, I’m not really in favor of this anonymity. I would have preferred a conversation with eye contact.” [Interview 14]	
	Lack of flexibility regarding IBI use	8 (20)	14	Participants perceived IBI use as inflexible as they felt tied down to a specific place to work with the IBI because of writing and reading on the computer, the internet connection requirements, and needing to sit in the office for long periods.		“The high effort to surf around on the Internet and that I could only do it here in the office.” [Interview 22]	
	Insufficient video and audio messages	6 (15)	7	The number of video and audio messages was perceived as limited or the content of the video and audio messages was perceived as unappealing.		“That there are more video or audio messages.... Just that I don’t have to read it, yes?! That it would have been more like watching TV, then it would have been even better, you know?!” [Interview 31]	
	Too few reminder emails	4 (10)	5	Participants found there were too few prompts or reminder emails with requests to complete the IBI.		“Maybe I would have needed more hints. Well, not hints, but prompts. It is perhaps sometimes annoying when you are reminded again and again, but I think that would have been helpful for me....” [Interview 38]	
**Intervention-related facilitators, IBI realization and design (n=5)^c^**							
	Independency regarding time	13 (33)	22	Participants perceived the option to participate in the IBI modules with flexibility of time (eg, opportunity to take a break and to cache) as helpful.		“What was suitable was that you could do it whenever you wanted. That you weren’t tied to certain times, I thought that was very good.” [Interview 11]	
	Flexible options in terms of IBI content	7 (18)	7	Participants found the flexibility to omit different topics of each IBI module (eg, tasks) or additional information if not needed to be helpful.		“There were always elective options.... There was something about ruminating thoughts or something else, where you had the choice, do you want to have some information on that, or not. I liked that, to be able to say in advance ‘No, I don’t need that now....’” [Interview 34]	
	Reminder emails	5 (13)	6	The regular reminder emails with prompts to continue the IBI were perceived as helpful.		“...but these reminders after a certain time, that was already quite good.” [Interview 6]	
	Appealing design and presentation	4 (10)	5	Participants perceived the design and presentation of the IBI as appealing.		“That it [the IBI] is very well presented, that practical, that it was very comprehensible.” [Interview 38]	
	Flexible IBI use from home	4 (10)	4	The option of flexibility to participate in the IBI modules from home and not needing to go to the city was perceived as helpful.		“And that’s why I find online training so valuable.... I don't have to get in the car and drive half an hour into town to get to therapy or anywhere else, and I don't have to shower beforehand. If need be, I can sit there in my stable clothes and go back to the stable afterwards....” [Interview 28]	

^a^Total number of excerpts, including multiple mentions from the same persons.

^b^Factors related to IBI realization and design (eg, composition, structure, and organization) that made it difficult to participate in the IBI.

^c^Factors related to IBI realization and design (eg, composition, structure, and organization) that made it easier to participate in the IBI.

**Table 5:** Major themes of the qualitative results for the work-related barriers and facilitators from participants’ perspectives (mentioned by at least 4 participants; N=40).

Categories		Participants			Definition		Supporting quotations
		Values, n (%)	Number of excerpts^a^				
**Work-related barriers (n=2)^b^**							
	Time-consuming work life	29 (73)	69	Participants experienced the tasks in everyday work life as time-intensive and inflexible because of weather influences, seasonal tasks, and work peaks and thus, as challenging for IBI^c^ participation.		“So actually, only operational work that is...very time-intensive and can’t be postponed...harvesting work or something like that, where you...say that HAS TO BE now. Now there is simply no time at all for three days.” [Interview 34]	
	Lack of staff leading to high workload	4 (10)	5	The (unforeseen) shortage of staff was experienced as aggravating for the workload, and thus, as challenging for IBI participation.		“This is a very special case, we don’t have an apprentice this year and so there’s a lack of manpower at all corners and then there’s the bad conscience again because the work doesn’t get done.” [Interview 17]	
**Work-related facilitators (n=1)^d^**							
	Flexible time management at work	25 (63)	34	Flexible time management at work (eg, because of self-employment, pension, lease of land, downsizing of the company, low workload, and season) made it easier to participate in the IBI.		“Yes, simply that you are self-employed, that you can arrange your work freely.” [Interview 20]	

^a^Total number of excerpts, including multiple mentions from the same persons.

^b^Factors related to the work life that made it difficult for the participants to take part in the internet-based intervention.

^c^IBI: internet-based intervention.

^d^Factors related to the work life that made it easier for the participants to take part in the internet-based intervention.

**Table 6:** Major themes of the qualitative results for the individual-related barriers and facilitators from participants’ perspectives (mentioned by at least 4 participants; N=40).

Categories (barriers)			Participants				Definition		Supporting quotations
			Values, n (%)		Number of excerpts^a^				
**Individual-related barriers (n=9)^b^**									
	Time-consuming private life	23 (58)		47		Participants perceived their private life as time-consuming because of household chores, hobbies, family, and friends, such that there was limited time available to work on the IBI^c^.		“That I was so busy privately, that I had no head for it. Primarily I had to take care of others to keep that going and myself I had to put aside. That was the only reason.” [Interview 38]	
	Lack of support from family and friends	17 (43)		32		Participants experienced family and friends (initially) as not being supportive, accepting, helpful, or motivating regarding their IBI participation.		“You know, now when I say I have an appointment with the family doctor, right?! To take blood. Then that’s alright. The environment knows that this has to be done now. But if I then sit down at the computer for an hour or ninety minutes and do something like that, well! Then...this is so negatively valued.” [Interview 31]	
	Limiting mental or cognitive factors	12 (30)		15		Limiting mental or cognitive factors (eg, exhaustion, tiredness, and difficulty in concentrating) were perceived as challenging.		“Either I was too tired or I had worked too much.” [Interview 36]	
	Lack of possibility of retreating to reflect on the IBI	10 (25)		12		Participants reported the lack of the possibility of retreating to a private and quiet space to reflect on the IBI as challenging.		“Predominantly it was a problem of time or just that there was too much hustle and bustle, so that you couldn’t really go back to it, or just actually hunkered down in a room where it was quiet.” [Interview 13]	
	Lack of computer skills or technical affinity	8 (20)		9		The lack of computer skills or technical affinity or the dislike of technical devices were perceived as challenging.		“That is quite concretely that I don’t really like to sit at the computer and don’t like to or rather would like to get away from surfing the Internet.” [Interview 29]	
	(Self-made) time or performance pressure	7 (18)		7		Participants experienced (self-made) time pressure or performance pressure (eg, regarding the IBI and regarding the job) as challenging.		“Yes, sometimes I didn’t progress as fast as I wanted, so I probably put myself under a bit of pressure there, but that had nothing to do with the training, because it’s the same for everyone....” [Interview 13]	
	Lack of perceived IBI success	6 (15)		12		Participants experienced no improvements because of the IBI (eg, no pain reduction or improvement in well-being) or reported that they did not consider the IBI to be promising for achieving improvements.		“...The decisive point was actually that...I thought that the training would be of no use to me...” [Interview 39]	
	Lack of motivation	6 (15)		6		Participants reported experiencing a lack of motivation or such a low level of psychological strain, that there was no motivation to work with the IBI.		“...Sometimes you’re just not motivated, let’s say you don’t feel like it or want to do something else, that you don't always want to deal with it. Yes, but then that’s a sign that you’re doing so well, that the pressure of psychological strain is no longer there....” [Interview 21]	
	Limiting somatic factors	4 (10)		6		Somatic factors (eg, chronic physical pain and pain caused by sitting for a long time) made it difficult to take part in the IBI.		“Yeah, because I’m in such massive pain and the painkillers didn’t work and then you can’t concentrate, not when there are so many, SO many questions that are actually always the same.” [Interview 11]	
**Individual-related facilitators (n=6)^d^**									
	Presence of motivation, curiosity, interest, and perseverance	30 (75)		39		Participants reported experiencing motivation, curiosity, and interest relating to the next modules or the feedback from the e-coach or referred to their own attitude to follow through on something that they started.		“Curiosity about the next lesson. And also, curiosity about the feedback from the e-coach.” [Interview 29]	
	Support from family and friends	20 (50)		46		The participants perceived support and acceptance from family and friends (eg, regarding the IBI use and regarding private and work life) as helpful.		“The family that has accepted everything and also notices, when you feel better or that you’re not so, let’s say, dissatisfied or whining, let’s say, so in that respect it’s already good now.” [Interview 15]	
	Perceived IBI success	19 (48)		34		Participants reported that it was helpful to observe improvements in everyday life, or to at least have the hope for success (eg, reduction of pain and improvement of well-being).		“The results I have felt for myself in my everyday life.” [Interview 22]	
	Flexible time management in private life	17 (43)		22		Flexible time management in private life (eg, because of living alone) was perceived as helpful.		“Yes, maybe...that the children are simply already more grown up. Well with small children, who then scream all the time, I don’t think that would have worked.” [Interview 20]	
	Scheduling of fixed time slots for the IBI	6 (15)		6		The scheduling of fixed time slots (eg, midday) for the IBI was perceived as helpful.		“Yes, I think it’s better if you just set certain...times, that you say, Monday evening at 8 or 9 pm I will do now one lesson” [Interview 20]	
	Possibility of retreating to reflect on the IBI	4 (10)		5		Participants reported the possibility of retreating to a private and quiet space to reflect on the IBI as helpful.		“...That I have my quiet, closed computer workplace. Where I have a place of retreat, so to speak, which is otherwise a workplace, but that I have used for this....” [Interview 21]	

^a^Total number of excerpts, including multiple mentions from the same persons.

^b^Factors related to the private life or personal factors that made it difficult for the participants to take part in the internet-based intervention.

^c^IBI: internet-based intervention.

^d^Factors related to the private life or personal factors that made it easier for the participants to take part in the internet-based intervention.

### Quantitative Follow-up Survey

In total, 73% (30/41) of the interview participants responded to the quantitative follow-up survey and rated whether they agreed with the barriers and facilitators that we had extracted from the interviews. Overall agreement with the identified barriers was relatively low, with a mean of 24% (SD 11%; range 7%-47%). At least 40% (12/30) of the participants agreed that the barriers (1) *extensive questioning*, (2) *missing key topics or unappealing focus*, (3) *IBI modules being too long*, (4) *the wish for continuing possibility to participate in follow-up modules or IBIs*, and (5) *lack of a platform for exchanges with other participants* hindered their participation in the IBI.

However, the agreement with the identified facilitators was very high, with a mean of 80% (SD, 13%; range 53%-97%). At least 90% (27/30) of the participants agreed that the factors (1) *possibility of working independently on the IBI modules*, (2) *independence regarding time*, (3) *flexible IBI use from home*, (4) *free-of-charge treatment offer*, (5) *appealing IBI structure and composition*, (6) *optimal organization*, and (7) *comprehensible wording* facilitated their participation in the IBI.

**Figure 1:**
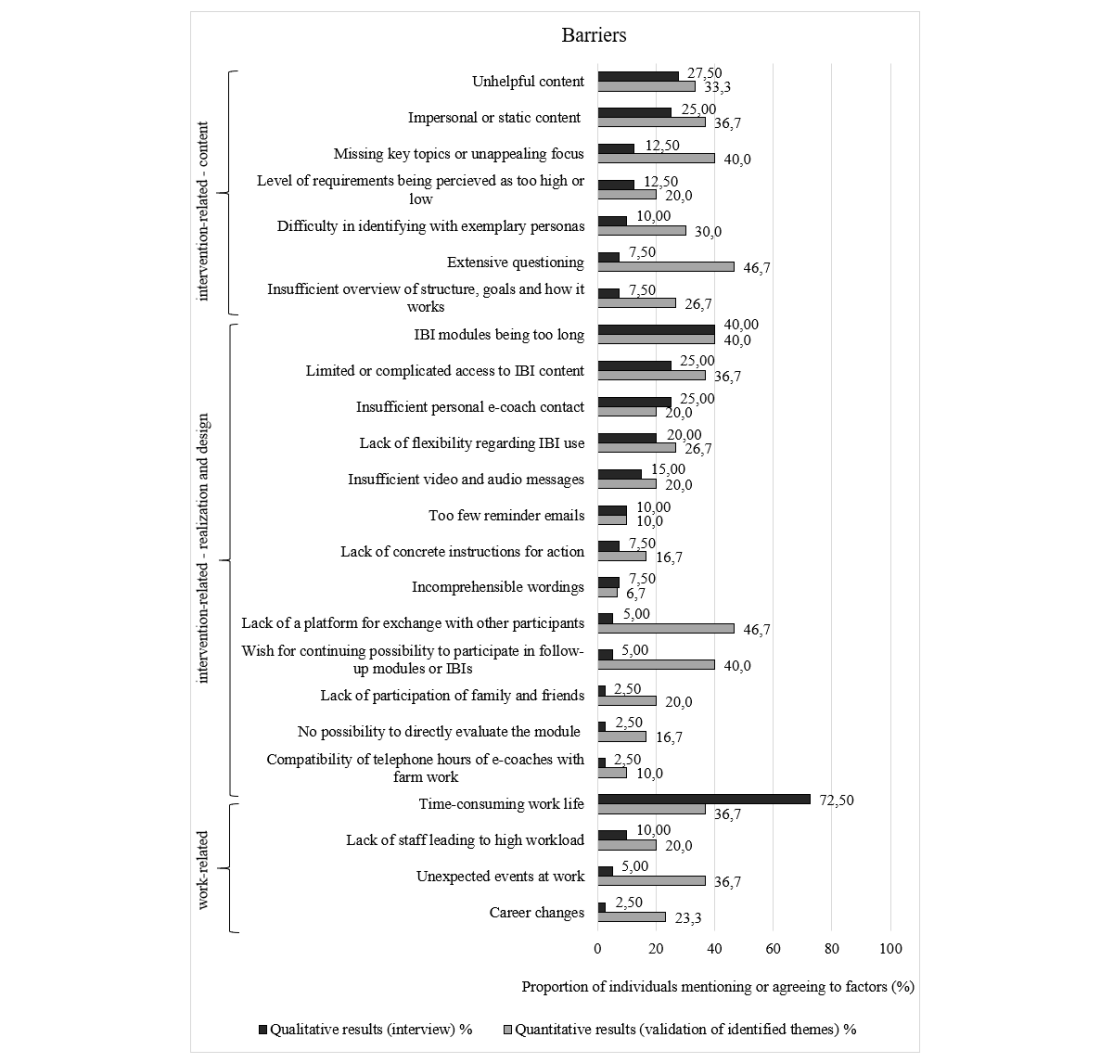
Comparison of quantitative and qualitative results regarding intervention- and work-related barriers. IBI: internet-based intervention.

**Figure 2:**
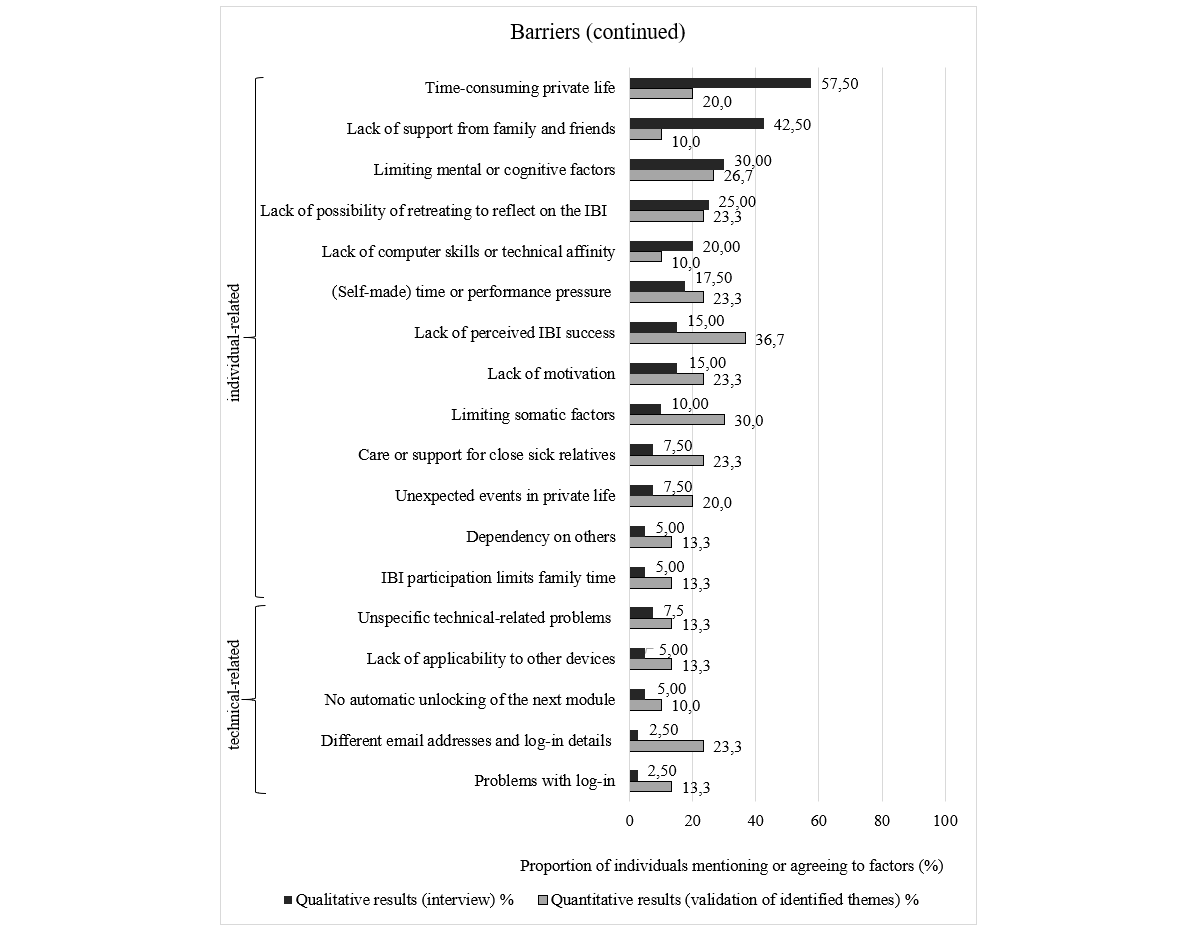
Comparison of quantitative and qualitative results regarding individual- and technical-related barriers. IBI: internet-based intervention.

**Figure 3:**
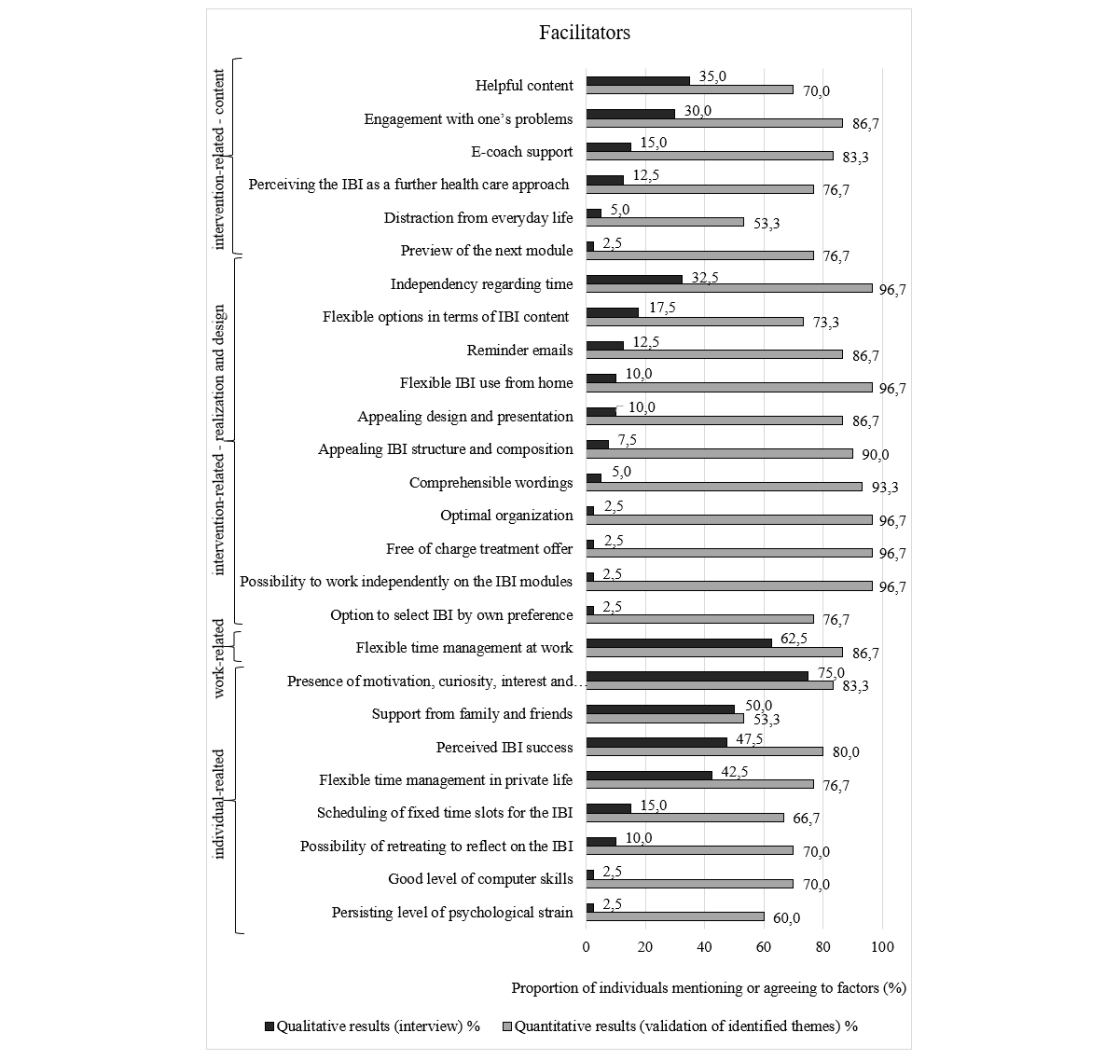
Comparison of quantitative and qualitative results regarding intervention-, work-, and individual-related facilitators. IBI: internet-based intervention.

Overall, the agreement rates for most of the identified barriers (32/42, 76%) and for all the facilitators were higher than the proportion of participants mentioning these in the interviews. Indeed, 27% (7/26) of facilitators that were mentioned by a single interview participant attained high agreement rates ranging between 60% (18/30) and 97% (29/30) in the follow-up survey. Figures 1-3 show the agreement rates in the quantitative follow-up survey for the barriers and facilitators compared with the number of participants mentioning these factors in the interviews.

### Subgroup Analysis Based on Quantitative Follow-up Survey

#### Subgroup Analysis for Completers and Noncompleters

**Figure 4:**
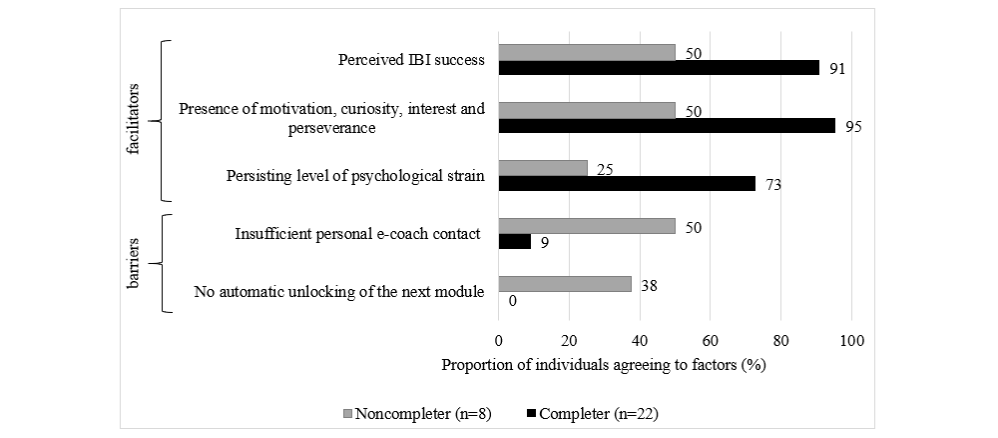
Significant group differences in perceived barriers and facilitators based on completer status in the quantitative follow-up survey (%). IBI: internet-based intervention.

A number of significant differences in agreement with relevant barriers and facilitators were found based on survey data, depending on the completer status. Noncompleters (8/30, 27%) reported agreement with the barrier *insufficient personal e-coach contact* (4/8, 50% vs 2/22, 9%; *P*=.03) and the technical-related barrier *no automatic unlocking of the next module* (3/8, 38% vs 0/22, 0%; *P*=.01) more often than completers (22/30, 73%). Completers reported agreement with the following factors as facilitating for training use significantly more often than noncompleters: having *perceived IBI success* or hope for IBI success (20/22, 91% vs 4/8, 50%; *P*=.03); having a personal attitude characterized by the *presence of* *motivation, curiosity, interest, and perseverance* with regard to training use (21/22, 95% vs 4/8, 50%; *P*=.01); and having a *persisting level of psychological strain* (16/22, 73% vs 2/8, 25%; *P*=.03). Figure 4 shows the relevant barriers and facilitators for completers versus noncompleters.

#### Subgroup Analysis for Different Treatment Indications

On the basis of a comparison of the different treatment indications leading to study inclusion, interviewees who received a pain-specific IBI (14/30, 47%), as indicated by their chronic pain symptomology (ie, PACT-A), agreed with having technical difficulties more often than interviewees who received a symptom-oriented tailored IBI (16/30, 53%) because of being at risk for depression (ie, PROD-A). This was specifically indicated by the barriers *dependency on others* regarding training use (4/14, 29% vs 0/16, 0%; *P*=.04), *problems with log-in* (4/14, 29% vs 0/16, 0%; *P*=.04), and *different email addresses and log-in details* (6/14, 43% vs 1/16, 6%; *P*=.03). Furthermore, *perceived IBI success* (16/16, 100% vs 8/14, 57%; *P*=.005) as facilitator was reported more often by interviewees who received a symptom-oriented tailored IBI for depression than by interviewees who received a pain-specific IBI. In addition, interviewees who received the pain-specific IBI agreed less often with *e-coach support* being facilitating (9/14, 64% vs 16/16, 100%; *P*=.01) for training use. Significant differences in agreement rates to barriers and facilitators among interviewees with different treatment indications are shown in Multimedia Appendix 4. Descriptive statistics and Fisher exact test results for all comparisons can be found in Multimedia Appendix 5.

## Discussion

### Principal Findings

To the best of our knowledge, this is the first study to investigate barriers and facilitators for the use of and adherence to IBIs among interviewees working in green professions. Using qualitative content analysis, we were able to identify a wide range of possible barriers and facilitators with regard to a tailored intervention approach taking into account 7 different symptom-specific IBIs and risk profiles for depression and pain interference. Overall, 42 barriers and 26 facilitators were identified across 4 superordinate categories relating to interventional, work-related, individual, or technical aspects. Insights were reached regarding the comparison of barriers and facilitators perceived in completers and noncompleters in particular.

### Comparison With Prior Work

Qualitative content analysis suggested that time restrictions in work and private life were elementary barriers from the interviewees’ perspective. This is consistent with a systematic review (3 qualitative and 3 quantitative studies) describing lack of time as a key barrier to adherence to IBIs for various psychological conditions (eg, coping with tinnitus, bipolar disorder, and unipolar depression) and target groups (eg, carers of persons with cancer and persons affected by disasters) [19]. Furthermore, lack of time was identified in different workplace settings as a barrier with regard to an internet intervention for stress management supported by optional guidance [20] as well as for the use of an internet-based depression prevention program with participants who were at risk for major depression [22]. As interviewees strongly agreed with barriers, such as extensive questioning and IBI modules being too long, this reflects the apparently high burden for some participants; thus, IBIs might require adjustments against the background of time restrictions in work and private life. The incompatibility of the time-consuming processing of extensive text contents with the personal situation of the participant was previously described as a potential factor for nonadherence in a qualitative study of nonadherers of an internet-based psychological treatment [59].

Nonetheless, a previous study reported that the use of IBIs seems to be associated with fewer barriers such as time constraints than participation in face-to-face treatment [60]. In farming populations, work life can be especially time-consuming depending on the season, which negatively affects the capacity for mental health help seeking [61,62]. Thus, technology-based alternatives have been suggested as low-threshold alternatives to facilitate mental health help seeking in farmers [63] and in rural areas [64]. Indeed, some interviewees reported flexible IBI use from home or flexibility in terms of time as helpful for IBI use, as well as the possibility of working independently on the IBI modules. These facilitators, as well as flexible options in terms of IBI content and IBI selection based on one’s own preference, found exceptionally high approval in the follow-up survey. Furthermore, barriers, such as limited or complicated access to training content, the option to repeat individual training units, missing key topics, or unappealing focus, which have received high approval ratings, reflect the need for more flexibility regarding training length and content in the interview sample. This indicates the importance of autonomy of the participants in their training conduct, which was identified as an important factor for adherence to an internet-based depression treatment in a blended care setting in a previous qualitative study [65]. It might also be related to the need for a sense of control by being able to complete the IBI flexibly and to return as often as necessary, as reported in a qualitative study of completers of an internet-based depression treatment [21] and is also relatable from the perspective of therapists reporting limited customizability and individualization of IBIs to be a barrier in blended therapy for depression [66].

Furthermore, interviewees emphasized flexible time management at work as a key facilitator, highlighting a new aspect that, to our knowledge, has not been previously identified. Flexibility of intervention use has been reported as both a facilitating and a hindering factor in a workplace setting if prioritization of time fails or temporal and spatial boundaries between work and treatment get blurred [20]. As we interviewed entrepreneurs, contributing spouses and family members, or pensioners working in their own business, this facilitator might have a specific meaning for self- or family-employed persons in this occupational target group.

Moreover, perceived support from family and friends was suggested by interviewees as a key facilitator. This factor has been described previously in terms of sense of belonging being a motivating aspect for continuing a depression treatment in a blended-therapy setting [65]. In traditional farming, family relationships play an important role, as farming families live and work together on joint premises [27]. Accompaniment by family has been shown to substantially increase health care use in farm workers [67], underlining the importance of family support against the background of lower use of professional help regarding mental health problems among farmers compared with nonfarmers [68]. Thus, involving the entire farming family in an IBI might be beneficial for increasing adherence, specifically in this occupational group. This would be in line with a different health care initiative already targeting the entire farming family [69] and with the statement of the interviewees that they missed exchanges with other IBI participants, which, to our knowledge, was identified for the first time in IBI research. Nevertheless, this seems to be in line with data from male farmers who reported seeking informal support from close confidants for self-help [70]. However, as some participants reported lack of support from family and friends, involving the farming family or close friends may not be indicated in every case and might also be a potential stressor.

Interviewees suggested the presence of motivation, curiosity, interest, and perseverance in using the IBI as another key facilitator for intervention completion. Similarly, interest in an IBI and willingness, and motivation to participate in it have previously been identified as facilitating factors by psychotherapists in a blended depression treatment [66]. Motivational and volitional aspects have been proposed as prerequisites for the uptake of and adherence to IBIs based on the Health Action Process Approach model that describes their central role in health behavior change [71]. So far, a systematic review based on qualitative data found mixed results regarding motivation and readiness to change as potential predictors of intervention adherence [19].

Against the background of low overall adherence rates in green professions for the IBIs in question [35,36] as an example of the actual use of IBIs in a pragmatic setting, a comparison of intervention completers and noncompleters regarding their agreement rates to the identified barriers and facilitators was conducted. The comparison analysis revealed that completers agreed significantly more often with the aspect of hope for or perceived training success. In the literature, this factor has already been described as noticing an improvement [21] or having hope of recovery [65] as facilitating persistence with the intervention. The affirmed motivational aspects in completers are in line with the previously discussed theoretical assumption that motivational factors such as interest and willingness to persist seem likely to drive adherence to IBI. Regarding the noncompleters, they agreed significantly more often that the e-coach contact was not personal enough. This mirrors the importance of the role of a stable therapeutic relationship in training adherence, as shown in previous qualitative studies [20,21,65,72]. A qualitative interview study with Australian farmers, their partners, and general practitioners suggested that a good relationship with health care professionals might be critical for the uptake of and adherence to treatment protocols and that for this to happen, it may be crucial that health professionals are agriculturally literate and able to personalize farmers' care through practical advice [63]. This has also been described as the lack of “farm credibility” of service providers being a barrier for the use of mental health services in farmers [62]. As IBIs were already adapted in an initial step in terms of content and design to the agricultural setting, further steps might comprise more participant inclusion in further adaptation of intervention design and content, as already practiced in an Australian IBI for farmers conveying mental health and well-being strategies based on acceptance and commitment therapy [73], or the offering of special training courses for e-coaches working in this occupational setting to improve “farm credibility.”

In addition, we compared interviewees included in the main trial because of chronic pain (ie, PACT-A) with interviewees included in the main trial because of psychological complaints indicating depression risk (ie, PROD-A) in terms of their perceived barriers to and facilitators for the use of their indicated IBI to determine potential differences. Interviewees included because of chronic pain agreed to experiences of training success or e-coach support being helpful less often than interviewees included because of being at risk for depression. As the presence of chronic pain symptoms is the main difference between these 2 interviewed groups, known factors such as high treatment resistance and long-term chronic pain symptomology [74] might be a possible explanation. Furthermore, a comparison of agreement rates in the follow-up survey suggested that interviewees included because of chronic pain reported technical difficulties and dependency on others as barriers for training use more often. This might be associated with the significantly higher average age of interviewees in PACT-A compared with PROD-A, as previous research has already shown that digital literacy tends to be higher in younger age groups [75]. Similarly, low digital literacy along with poor connectivity in rural areas was identified as a barrier to the use of IBIs among Australian farmers based on an interview study [63]. Overall, the interview results suggest that digital literacy may be restricted to some of the interviewees at hand. To our knowledge, no research exists pertaining to the extent of digital literacy in persons occupied in green professions in Germany in general, despite the face validity of the assumption that digital literacy might be lower in the green sector than in the general population. This would be in line with a recent article suggesting that digital literacy may be lower in rural areas than in urban areas [76]. At least a few years ago, limited internet literacy and access were reported as being among the most important barriers to the implementation of IBIs for depression in routine care expected from the perspective of different stakeholders across 8 European countries [77]. Lack of internet access and computers along with a lack of familiarity with technology, internet, and media were reported as barriers from the perspective of an interview sample of general practitioners [78] as well as low technical affinity from the perspective of an interview sample of therapists, both with regard to the use of blended internet-based therapy for patients with depression in general [66]. Thus, improving access to high-speed internet in rural or remote areas along with the promotion of digital literacy in general might be helpful measures to break down barriers in the use of health care technologies in green professions.

### Limitations and Strengths

This mixed methods study has several limitations. First, the interview sample was not representative of the population of green professions. We aimed to recruit a heterogeneous interview sample taking into account sex, occupational role, IBI type, and completer status to achieve the best representativeness possible. However, the self-selection of the participants regarding interview and survey participation was evident, as the sample of noncompleters was reduced from 41% (17/41) in the interview sample to 27% (8/30) in the follow-up survey. Thus, there is a noticeable underrepresentation of noncompleters in both RCTs, where approximately every second intervention participant at the 12-month follow-up did not complete the intervention (PROD-A: 85/171, 50%; PACT-A: 23/42, 55%). Overall, this might have led to a bias regarding the results of qualitative data analysis in favor of possibly fewer reported barriers. Furthermore, this might have favored an overestimation of the approval rates regarding the identified facilitators in the follow-up survey. Indeed, the approval rates of the identified facilitators were higher than those of the identified barriers, even though considerably more barriers than facilitators were identified overall. Second, the subgroup analyses performed were of purely explorative character, as there were no a priori hypotheses defined, there was no power calculation conducted beforehand, and the sample size of 30 was very small with regard to quantitative analyses. Thus, these subgroup analyses merely provide an opportunity to gain an idea of possible factors influencing the engagement with and adherence to IBIs in this specific occupational group. Further studies need to be conducted based on a priori power calculations to systematically investigate the identified barriers and facilitators as predictors of engaging with and adhering to IBIs. Third, there is possibly a bias because of the inclusion of interviewees from 2 different study populations with different treatment indications, and thus, a bias because of the overrepresentation of interviewees who received GET.ON Chronic Pain compared with those who received other IBI types. This might have facilitated a stronger focus on barriers and facilitators specific to the use of this IBI experienced by participants with chronic pain symptoms in comparison with those at risk for depression. Therefore, we carried out a comparative analysis of these 2 subpopulations to unravel and highlight potential differences in perceived barriers to and facilitators for the use of their corresponding IBIs because of different treatment indications and underlying symptoms of primarily somatic versus mental nature.

This study has also several strengths. First, the coded interview material was exceptionally extensive, consisting of 40 interviews overall, and thus, allowed for a comprehensive identification of possible barriers and facilitators. Second, the purposeful sampling procedure and the resulting heterogeneity of the interview sample enabled us to identify a wide range of barriers and facilitators of potential relevance. Owing to increased recruitment efforts, the perspective of noncompleters, in particular, could be taken into account. This may have resulted in the identification of substantially more barriers than facilitators, based on the qualitative interviews. Against the background of limited intervention adherence experienced in this occupational group [35,36], these results provide first insights into possible obstacles and enable to derive implications for facilitating intervention adherence. Third, the intercoder reliability was exceptionally high, reflecting the high methodological standards of the qualitative coding procedure. Fourth, by using a mixed methods approach, we were able to conduct explorative quantitative comparisons among different subgroups to evaluate possible divergences in the identified barriers and facilitators depending on completer status or treatment indication. Thus, this approach enabled us to achieve a more differentiated view of barriers and facilitators for training use and an idea about the possible relevance of individual factors.

### Conclusions

Different implications for promoting green profession workers’ engagement with and adherence to IBIs are imaginable based on the findings of this study. On the basis of the insights on the facilitators, we can conclude that the following factors pertaining to the IBIs worked particularly well from the perspective of the interview sample: (1) flexible use independent of time and location, (2) flexible options in terms of IBI content, (3) appealing design and presentation, (4) appealing structure and composition, (5) optimal organization, (6) overall helpful content, and (7) support of the e-coach. We derived the following options to further improve the use of IBIs in the occupational group of green professions based on insights into the identified barriers: the implementation of (1) supportive strategies like the scheduling of fixed time slots for the IBI or tools such as push messages to facilitate training completion; (2) options to involve the entire farming family or friends and colleagues and to enable exchanges between participants, for example, via implementation of a forum, chat functions, or even group sessions with the e-coach; or (3) enabling face-to-face interaction with the e-coach via video chat on demand. Further ideas encompass (4) the customization of intervention modules in terms of intervention content and length to allow for more flexible and adaptive use or (5) the introduction of the option to flexibly access and repeat specific content. Thus, in future studies, these factors should be compared with existing theoretical frameworks and then jointly investigated using quantitative study designs and methods in terms of their ability to improve intervention uptake and adherence in the specific target group of green professions.

## Appendix

Multimedia Appendix 1 COREQ (Consolidated Criteria for Reporting Qualitative Research) checklist.

Multimedia Appendix 2 Comparison of participant characteristics of the interview subsamples in terms of study affiliation and intervention arms of the randomized controlled trials Prevention of Depression in Agriculturists (PROD-A) and Preventive Acceptance and Commitment Therapy for Chronic Pain in Agriculturists (PACT-A).

Multimedia Appendix 3 Overview of the minor themes of the qualitative results for the identified barriers and facilitators (each mentioned by fewer than 4 participants).

Multimedia Appendix 4 Significant differences in barriers and facilitators between Prevention of Depression in Agriculturists (PROD-A) and Preventive Acceptance and Commitment Therapy for Chronic Pain in Agriculturists (PACT-A) interviewees based on the agreement rates in the quantitative follow-up survey.

Multimedia Appendix 5 Descriptive statistics and Fisher exact test results of subgroup comparisons pertaining to completer status and study affiliation based on the agreement rates for the identified barriers and facilitators in the quantitative follow-up survey.

## Abbreviations

COREQ: Consolidated Criteria for Reporting Qualitative Research
IBI: internet-based intervention
PACT-A: Preventive Acceptance and Commitment Therapy for Chronic Pain in Agriculturists
PROD-A: Prevention of Depression in Agriculturists
RCT: randomized controlled trial
