# Supplementary material for: Barriers to and Facilitators of Engaging With and Adhering to Guided Internet-Based Interventions for Depression Prevention and Reduction of Pain-Related Disability in Green Professions: Mixed Methods Study
Source: JMIR Ment Health. 2022 Nov 9;9(11):e39122. doi: 10.2196/39122 (PMC9685507; doi:10.2196/39122)
Supplement: Multimedia Appendix 1 [file mental_v9i11e39122_app1.docx]

| **Multimedia Appendix 1.**  Consolidated criteria for reporting qualitative studies (COREQ): 32-item checklist. | | |
| --- | --- | --- |
| **No. Item** | **Guide questions/description** | **Reported** |
| **Domain 1: Research team and reﬂexivity** | | |
| *Personal Characteristics* | | |
| 1. Interviewer/ facilitator | Which persons conducted the interviews or focus groups? | Interviews of PROD-A participants were conducted by Gasde, Manuela (MG) and Riedel, Andrea (AR). Interview conduct of PROD-A interviews was supervised by researcher Freund, Johanna (JF). Interviews of PACT-A participants were conducted by Locker, Saskia (SL). Interview conduct of PACT-A interviews was supervised by researcher Braun, Lina (LB). |
| 2. Credentials | What were the interviewer’s credentials? *E.g. PhD, MD* | Bachelor of Science |
| 3. Occupation | What was their occupation at the time of the study? | Master student |
| 4. Gender | Was the researcher male or female? | Female |
| 5. Experience and training | What experience or training did the interviewer have? | Interviewers had no prior experience and were trained by conducting an interview in role play with feedback by the supervisors |
| *Relationship with participants* | | |
| 6. Relationship established | Was a relationship established prior to study commencement? | No relationship was established prior to interview study. The first contact was made prior to interview conduct. |
| 7. Participant knowledge of the interviewer | What did the participants know about the researcher? *e.g. personal goals, reasons for doing the research* | No information about tasks and responsibilities of the researchers was shared. No personal goals. Reasons for interview conduct were stated at the beginning of the interview. |
| 8. Interviewer characteristics | What characteristics were reported about the interviewer/facilitator? *e.g. Bias, assumptions, reasons and interests in the research topic* | No personal characteristics were reported about the interviewer/facilitator |
| **Domain 2: study design** | |  |
| *Theoretical framework* | |  |
| 9. Methodological orientation and Theory | What methodological orientation was stated to underpin the study? *e.g. grounded theory, discourse analysis, content analysis* | Qualitative content analysis |
| *Participant selection* | |  |
| 10. Sampling | How were participants selected? *e.g. purposive, convenience, consecutive, snowball* | Purposeful theoretical sampling, taking participant characteristics (particularly gender, occupational role, completer status, type of IBI) into account, interviewees were selected from intervention groups of two RCT trials |
| 11. Method of approach | How were participants approached? *e.g. face-to-face, telephone, email* | All participants were approached initially by e-mail. PROD-A participants were reminded via e-Mail, whereas PACT-A participants were additionally contacted via telephone due to the small sample size |
| 12. Sample size | How many participants were in the study? | *N*=22 interviews were conducted with intervention participants from PROD-A,  *N*=19 interviews were conducted with intervention participants from PACT-A |
| 13. Non-participation | How many people refused to participate or dropped out? Reasons? | None |
| *Setting* |  |  |
| 14. Setting of data collection | Where was the data collected? *e.g. home, clinic, workplace* | Data was collected via telephone and participants were able to participate from anywhere. Data collection for PACT-A interviews was conducted throughout July to August 2019, for PROD-A interviews throughout September to November 2019. |
| 15. Presence of non-participants | Was anyone else present besides the participants and researchers? | No |
| 16. Description of sample | What are the important characteristics of the sample? *e.g. demographic data, date* | Interviews were conducted with entrepreneurs, supporting spouses and family members or pensioners occupied in green professions (i.e. agriculture, forestry, horticulture) at risk for depression (PROD-A participants) or suffering from chronic pain (PACT-A participants) |
| *Data collection* |  |  |
| 17. Interview guide | Were questions, prompts, guides provided by the authors? Was it pilot tested? | Questions, prompts and guides were provided in the interview guide. The first interview with PACT-A interviewees and the first two interviews with PROD-A interviewees were used as pilot interviews. Pilot interviews were discussed with supervisors, minor changes were made to the interview guide after the first pilot interview, pilot interviews were included in data analysis |
| 18. Repeat interviews | Where repeat interviews carried out? If yes, how many? | No repeat interviews |
| 19. Audio/visual recording | Did the research use audio or visual recording to collect the data? | Audio recording only (PROD-A: PhonerLite and Sipgate, PACT-A: voice recorder). PROD-Interviews were transcribed by MG, AR; PACT-A interviews were transcribed by SL and Pausch, Sophie (SP) |
| 20. Field notes | Were field notes made during and/or after the interview or focus group? | Field notes were only made for PROD-A interviewees |
| 21. Duration | What was the duration of the interviews or focus group? | PROD-A interviews: mean 10 min (SD 3; range 6-19); PACT-A interviews: mean 9 min (SD 3; range 5-19) |
| 22. Data saturation | Was data saturation discussed? | Yes, data saturation was discussed and concluded as reached |
| 23. Transcripts returned | Were transcripts returned to participants for comment and/or correction? | No |
| **Domain 3: analysis and ﬁndings** | |  |
| *Data analysis* |  |  |
| 24. Number of data coders | How many data coders coded the data? | Two coders: SP and Beywl, Lea, (LBE). The coding procedures were supervised by researcher LB. |
| 25. Description of the coding tree | Did authors provide a description of the coding tree? | Yes |
| 26. Derivation of themes | Were themes identiﬁed in advance or derived from the data? | We used an inductive-explorative approach and derived themes from the data |
| 27. Software | What software, if applicable, was used to manage the data? | MAXQDA (Version 2018.2) was used for transcription and qualitative data analysis |
| 28. Participant checking | Did participants provide feedback on the ﬁndings? | Yes, identified categories were presented to the participants for agreement to validate the results |
| *Reporting* |  |  |
| 29. Quotations presented | Were participant quotations presented to illustrate the themes / findings? Was each quotation identified? *e.g. participant number* | Yes |
| 30. Data and ﬁndings consistent | Was there consistency between the data presented and the ﬁndings? | Yes |
| 31. Clarity of major themes | Were major themes clearly presented in the ﬁndings? | Yes |
| 32. Clarity of minor themes | Is there a description of diverse cases or discussion of minor themes? | Yes |
| Note. Tong A, Sainsbury P, Craig J. Consolidated criteria for reporting qualitative research (COREQ): a 32-item checklist for interviews and focus groups. International Journal for Quality in Health Care. 2007. Volume 19, Number 6: pp. 349 – 357. | | |
