# Supplementary material for: Barriers to and Facilitators of Engaging With and Adhering to Guided Internet-Based Interventions for Depression Prevention and Reduction of Pain-Related Disability in Green Professions: Mixed Methods Study
Source: JMIR Ment Health. 2022 Nov 9;9(11):e39122. doi: 10.2196/39122 (PMC9685507; doi:10.2196/39122)
Supplement: Multimedia Appendix 2 [file mental_v9i11e39122_app2.docx]

**Multimedia appendix 2.**

**Table 1.** Characteristics of the interview sample compared to intervention arms of PROD-A and PACT-A.

|  |  | All interview participants (n=41) | Interview participants PROD-A^a^ (n=22) | Total IG^b^ PROD-A (n=171) | Interview participants PACT-A^c^ (n=19) | Total IG PACT-A (n=43) | t test (*df*)^d^ | *P* value^e^ |
| --- | --- | --- | --- | --- | --- | --- | --- | --- |
| **Sociodemographic characteristics** | | |  |  |  |  |  |  |
|  | Sex (male), n (%) | 17 (41) | 11 (50) | 69 (40) | 6 (32) | 13 (30) | N/A | .34 |
|  | Age (years), mean (SD) | 55.88 (7.86) | 53.27 (8.14) | 50.02 (9.58) | 58.89 (6.49) | 57.23 (9.50) | 2.42 (39) | .02 |
|  | In a partnership or married, n (%) | 39 (95) | 20 (91) | 154 (90) | 19 (100) | 41 (95) | N/A | .49 |
| **Education, n (%)** | |  |  |  |  |  |  | .27 |
|  | Low | 18 (44) | 7 (32) | 73 (43) | 11 (58) | 28 (65) | N/A |  |
|  | Middle | 13 (32) | 8 (36) | 49 (29) | 5 (26) | 9 (21) | N/A |  |
|  | High | 10 (24) | 7 (32) | 49 (29) | 3 (16) | 6 (14) | N/A |  |
| **Occupational role, n (%)** | | |  |  |  |  |  | .25 |
|  | Entrepreneur | 16 (39) | 10 (45) | 91 (53) | 6 (32) | 15 (35) | N/A |  |
|  | Contributing spouse | 12 (29) | 6 (27) | 48 (28) | 6 (32) | 12 (28) | N/A |  |
|  | Contributing family member | 5 (12) | 3 (14) | 17 (10) | 2 (11) | 7 (16) | N/A |  |
|  | Pensioner or spouse of pensioner | 6 (15) | 1 (5) | 11 (6) | 5 (26) | 9 (21) | N/A |  |
|  | Incapacitated for work | 2 (5) | 2 (9) | 4 (2) | 0 (0) | 0 (0) | N/A |  |
| **Adherence to IBI^f^, n (%)** | | |  |  |  |  |  | .54 |
|  | Completer (100% modules) | 24 (59)^g^ | 14 (64)^g^ | 86 (50)^h^ | 10 (53)^g^ | 19 (45)^h,i^ | N/A |  |
|  | Noncompleter (<100% modules) | 17 (41)^g^ | 8 (36)^g^ | 85 (50)^h^ | 9 (47)^g^ | 23 (55)^h,i^ | N/A |  |
| **Type of IBI, n (%)** | |  |  |  |  |  |  | <.001 |
|  | GET.ON Mood Enhancer | 5 (12) | 5 (23) | 42 (25) | N/A | N/A | N/A |  |
|  | GET.ON Stress | 11 (27) | 11 (50) | 103 (60) | N/A | N/A | N/A |  |
|  | GET.ON Recovery | 2 (5) | 2 (9) | 15 (9) | N/A | N/A | N/A |  |
|  | GET.ON Panic | 1 (2) | 1 (5) | 5 (3) | N/A | N/A | N/A |  |
|  | GET.ON Be clever - drink less | 2 (5) | 2 (9) | 2 (1) | N/A | N/A | N/A N/A |  |
|  | GET.ON Mood Enhancer Diabetes | 1 (2) | 1 (5) | 2 (1) | N/A | N/A | N/A |  |
|  | GET.ON Chronic Pain | 19 (46) | N/A | N/A | 19 (100) | 43 (100) | N/A |  |
|  | No training assignment | 0 (0.0) | 0 (0) | 2 (1) | N/A | N/A | N/A |  |
| **Period between baseline and interview (months), n (%)** | | | | |  |  |  | .34 |
|  | < 6 | 2 (5) | 0 (0) | N/A | 2 (11) | N/A | N/A |  |
|  | 6-12 | 14 (34) | 7 (32) | N/A | 7 (37) | N/A | N/A |  |
|  | > 12 | 25 (61) | 15 (68) | N/A | 10 (53) | N/A | N/A |  |

^a^PROD-A: Prevention of Depression in Agriculturists.

^b^IG: Intervention group.

^c^PACT-A: Preventive Acceptance and Commitment Therapy for Chronic Pain in Agriculturists.

^d^t Test was used only for continuous variables.

^e^*P* value is based for continuous variables on a t-test, for categorical variables on an exact Fisher’s test and is calculated for comparison between interview participants from PROD-A and PACT-A.

^f^IBI: internet-based intervention.

^g^for interview participants: adherence to IBI is reported at the time of interview conduct. Adherence to IBI has been compared for each interviewed person at time of interview conduct and at 12-months follow-up. There were no changes of category regarding categories “completer”/”non-completer”.

^h^for total intervention arms of PROD-A and PACT-A: adherence to IBI is reported at 12-month follow-up.

^i^sample for intervention variables correspond to n=42 as one participant requested deletion of data on intervention platform.
