# Supplementary material for: Barriers to and Facilitators of Engaging With and Adhering to Guided Internet-Based Interventions for Depression Prevention and Reduction of Pain-Related Disability in Green Professions: Mixed Methods Study
Source: JMIR Ment Health. 2022 Nov 9;9(11):e39122. doi: 10.2196/39122 (PMC9685507; doi:10.2196/39122)
Supplement: Multimedia Appendix 3 [file mental_v9i11e39122_app3.docx]

**Multimedia Appendix 3.**

**Table 1.** Minor themes of the qualitative results for the intervention-related barriers and facilitators pertaining to internet-based intervention (IBI) content from participants’ perspectives (mentioned by less than 4 participants; N=40).

|  | Participants | | Definition | Supporting quotations |
| --- | --- | --- | --- | --- |
|  | Values, n (%) | Number of excerpts^a^ |  |  |
|  | | | | |
| **Intervention-related barriers, IBI content (n=2)^b^** | | | | |
| Extensive questioning | 3 (8) | 5 | Some participants experienced the questions in each module as too extensive and repetitive. | “(…) the many long, awkward questions, the filling out of the booklets and then you forgot something and then it went back to the page (…), that was already a bit tedious. It took a lot of discipline (…)” [Interview 21] |
| Insufficient overview of structure, goals, and how it works | 3 (8) | 4 | Some participants wished a priori for a clearer overview and structure of the IBI and experienced difficulties in understanding how the IBI was supposed to be applied and to help for the individual problem. | "Maybe a clearer outline. For the whole structure. So that maybe I would have also understood what to do BESIDES the question." [Interview 33] |
| **Intervention-related facilitators, IBI content (n=2)^c^** | | | | |
| Distraction from everyday life | 2 (5) | 2 | To see the IBI as a distraction or pause from everyday life was helpful. | “I say for many maybe this is a distraction from everyday life.” [Interview 4] |
| Preview of the next module | 1 (3) | 1 | The short preview at the end of each module of the topic and content of the next module made it easier to continue. | "At the end of the lesson, yes, there was a little preview what was going to come in the next lesson, and that was a little bit of, yeah, a taste, and that's when you got curious to stay tuned." [Interview 13] |

^a^Total number of excerpts including multiple mentions from the same persons.

^b^Factors related to the IBI content (eg, specific exercises), that made it difficult to participate in the IBI.

^c^Factors related to the IBI content (eg, specific exercises), that made it easier to participate in the IBI.

**Table 2.** Minor themes of the qualitative results for the intervention-related barriers and facilitators pertaining to internet-based intervention (IBI) realization and design from participants’ perspectives (mentioned by less than 4 participants; N=40).

| Categories | Participants | | Definition | Supporting quotations |
| --- | --- | --- | --- | --- |
|  | Values, n (%) | Number of excerpts^a^ |  |  |
|  | | | | |
| **Intervention-related barriers, IBI realization and design (n=7)^b^** | | | | |
| Incomprehensible wordings | 3 (8) | 5 | Some participants perceived the wording used as too theoretical, incomprehensible, and not colloquial enough, thus the IBI content was reported as difficult to understand. | "Yeah, the wording was already sometimes very, yeah, that you had to read it two or three times before you knew exactly what they even wanted." [Interview 11] |
| Lack of concrete instructions for action | 3 (8) | 5 | Some participants found that the instructions in the IBIs were not concrete enough or that instructions for specific topics they had expected were missing at all. | "Well, just short and to the point and factually structured. And with a direct approach: What do I have to do? Not with any case studies or not with any palaver and pictures of any people (...). Short, concise, factual and correct. What you have to do and that's that!" [Interview 33] |
| Lack of a platform for exchange with other participants | 2 (5) | 3 | The lack of a platform to enable exchange with other IBI participants was experienced as challenging. | "If I had known that there was a colleague who was also doing this, it would have been possible to briefly exchange ideas (…)” [Interview 13] |
| Wish for continuing possibility to participate in follow-up modules or IBIs | 2 (5) | 2 | Some participants expressed the wish for the possibility to take part in follow-up refresh IBI modules or have the possibility to participate in another IBI if need be. | "I would have to be able to sign up and say "I have such and such a problem and I would like to do a training like this again." [Interview 22] |
| Lack of participation of family and friends | 1 (3) | 2 | One of the participants experienced the lack of participation of family and friends in the IBI as challenging. | "But I would find it better if they ALSO participated. Because they also work on the farm. We actually have the same problems." [Interview 22] |
| Compatibility of telephone hours of e-coaches with farm work | 1 (3) | 1 | The lack of possibility to undertake the phone calls with the e-coach at hours that are well compatible with farmers work was experienced as challenging. | "But perhaps telephone-based at times that are convenient for farmers and winegrowers. To say, how about talking to each other for a quarter of an hour in the morning between six and seven o'clock and then we have some space. Or in the evening then starting at eight pm, then there is more quietness again." [Interview 40] |
| No possibility to directly evaluate the module | 1 (3) | 1 | One of the participants would have liked to directly express his opinion during the completion of the IBI module | “So, in the lessons I always had no possibility to express my personal opinion. I didn't find that so good. Because (…), what went better, what went worse, and one worked off the tasks, worked off, worked off and I would have liked to express my feedback right there.” [Interview 22] |
| **Intervention-related facilitating factors, IBI realization and design (n=6)^c^** | | | | |
| Appealing IBI structure and composition | 3 (8) | 5 | The IBI structure and composition of the IBI was perceived as appealing. | “Nope, from the structure I found it quite good.” [Interview 11] |
| Comprehensible wordings | 2 (5) | 2 | The wording used and thus, the IBI content was perceived as comprehensible and easy to understand. | “It was simple, it was comprehensible, it was interesting.” [Interview 26] |
| Optimal organization | 1 (3) | 1 | The organization of the IBI participation was experienced as smoothly and technically frictionless. | “From a technical and organizational point of view, let's say, I thought it was optimal the way it went.” [Interview 15] |
| Free of charge treatment offer | 1 (3) | 1 | The opportunity to use the IBI free of charge as an insurance benefit was experienced as a positive aspect. | “I am thrilled that the gardeners' health insurance fund offers something like this! It doesn't really have a good reputation otherwise. It's cheap, but you don't get much either.” [Interview 35] |
| Possibility to work independently on the IBI modules | 1 (3) | 1 | Participants experienced the possibility to work on the modules completely independently as helpful for IBI participation. | “As I was able to divide it up nicely in terms of time and subject matter, I was ultimately forced to do something on my own too, which was a good mix.“ [Interview 13] |
| Option to select IBI by own preference | 1 (3) | 1 | The consideration of participant preference in IBI selection made it easier to take part in the IBI. | “Because I have chosen the subject myself, panic.” [Interview 25] |

^a^Total number of excerpts including multiple mentions from the same persons.

^b^Factors related to the IBI realization and design (eg, composition, structure, and organization), that made it difficult to participate in the IBI.

^c^Factors related to the IBI realization and design (eg, composition, structure, and organization), that made it easier to participate in the IBI.

**Table 3.** Minor themes of the qualitative results for the work-related barriers and facilitators from participants’ perspectives (mentioned by less than 4 participants; N=40).

| Categories | Participants | | Definition | Supporting quotations |
| --- | --- | --- | --- | --- |
|  | Values, n (%) | Number of excerpts^a^ |  |  |
|  | | | | |
| **Work-related barriers (n=2)^b^** | | | | |
| Unexpected events at work | 2 (5) | 2 | Unforeseen, unplanned events at work (eg, spontaneous tasks, filling in for co-workers) interfered with IBI^c^ participation | "Or employees who have been absent, where I have had to step in, or sudden tasks that have occurred somewhere, so that I can't say "I'm off now." That doesn't always work." [Interview 22] |
| Career changes | 1 (3) | 1 | Changes in professional life (eg, sale of the business) were experienced as challenging for the IBI participation | "Yes, simply too much preoccupation with work or with what has to be done or what is going wrong, because there is a lot of upheaval going on in my case now, and that is simply often a hindrance, because it keeps you busy, and is very, very, very time-consuming." [Interview 8] |
| **Work-related facilitators (n=0)^d^** | | | | |

^a^Total number of excerpts including multiple mentions from the same persons.

^b^Factors related to the work life, that made it difficult for the participants to take part in the IBI.

^c^IBI: internet-based intervention.

^d^Factors related to the work life, that made it easier for the participants to take part in the IBI.

**Table 4.** Minor themes of the qualitative results for the individual-related barriers and facilitators from participants’ perspectives (mentioned by less than 4 participants; N=40).

| Categories | Participants | | Definition | Supporting quotations |
| --- | --- | --- | --- | --- |
|  | Values, n (%) | Number of excerpts^a^ |  |  |
|  | | | | |
| **Individual-related barriers (n=4)^b^** | | | | |
| Unexpected events in private life | 3 (8) | 4 | Unforeseen, unplanned events of the private life (eg, visits, accidents, spontaneous activities) interfered with IBI^c^ participation. | "(…) Since I often did it on the weekend, there were maybe visitors who came who were not planned or that we spontaneously went somewhere else. So actually, exactly that where this online training also supports me. Like doing positive things." [Interview 35] |
| Care or support for close sick relatives | 3 (8) | 3 | A nursing case in the family or the support of close relatives who fell ill and were unable to contribute to the family business made it difficult to participate in the IBI | "Yes, the circumstances that I still have to cope with in my private life, i.e. the care, the nursing case and everything that is connected with it. When you are confronted with the nursing case from one moment to the next and then still have the business, where everything has to be handled, you can't always be available for private things. And this training was something private." [Interview 7] |
| Dependency on others | 2 (5) | 3 | Participants experienced the dependency on others to be able to work on the IBI on the computer as challenging. | "Yes, I'm saying that the kids set it up and when I got stuck on the computer then I could also fall back on the kids again, but then again this was very difficult. Then I called them and then they had to come back over again and help me out." [Interview 7] |
| IBI participation limits family time | 2 (5) | 2 | Participants experienced as challenging that they could spend less time with their family due to their IBI participation | "(…) when I finish work, I have time for my family, which I don't have in that case, because I've been sitting at the computer or on the iPad." [Interview 14] |
| **Individual-related facilitators (n=2)^d^** | | | | |
| Good level of computer skills | 1 (3) | 1 | A good level of computer skills was perceived as helpful. | “And I have little inhibition about participating in such an online offer, because I'm actually already quite fit in dealing with computers and the Internet and so on.” [Interview 34] |
| Persisting level of psychological strain | 1 (3) | 1 | The persisting level of psychological strain (e.g. chronic pain) increased the motivation to continue the IBI participation | “Yes, because if you have a pain again and again, then you think about it again. (…) So the physical constellation, let's say so.” [Interview 27] |

^a^Total number of excerpts including multiple mentions from the same persons.

^b^Factors related to the private life or personal factors, that made it difficult for the participants to take part in the IBI.

^c^IBI: internet-based intervention.

^d^Factors related to the private life or personal factors, that made it easier for the participants to take part in the IBI.

**Table 5.** Minor themes of the qualitative results for the technical-related barriers and facilitators from participants’ perspectives (mentioned by less than 4 participants; N=40).

| Categories | Participants | | Definition | Supporting quotations |
| --- | --- | --- | --- | --- |
|  | Values, n (%) | Number of excerpts^a^ |  |  |
|  | | | | |
| **Technical-related barriers (n=5)^b^** | | | | |
| Unspecific technical-related problems | 3 (8) | 5 | Participants reported unspecific technical-related problems with either the necessary devices or processes that made it difficult to take part in the IBI^c^. | “Yes, as I already said, sometimes it was already late in the evening and it didn’t work right, that hindered me.” [Interview 37] |
| No automatic unlocking of the next module | 2 (5) | 10 | Participants experienced that the next IBI module could not be accessed automatically [*note: as participants failed to click on “send” after completion of the IBI module*] and they could not continue with the IBI. | “I didn’t have to drop out of the training, I just didn’t receive any more tasks from you.” [Interview 12] |
| Lack of applicability to other devices | 2 (5) | 3 | Participants reported the wish to have the possibility complete small exercises or the entire IBI on other devices (tablet, smartphone). | “Well, to manage to make the thing work via the tablet, as I think that would be helpful and easier for many people, I guess. (…)” [Interview 8] |
| Problems with log-in | 1 (3) | 2 | Participants reported problems with the log-in on a regular basis that made it difficult to participate in the IBI | “And if I go back in now, I have a communication in GET.ON now and I can't get in there, it doesn't work. I can't get in, I have two passwords here, where I try, but it doesn't work. (...)” [Interview 12] |
| Different email addresses and log-in details | 1 (3) | 2 | The use of different email addresses and log-in details for different platforms (GET.ON, minddistrict, study team) was perceived as confusing | “(…) I have the study team "With us in balance", then I have [e-mail address], then I have [intervention platform], I have different e-mail addresses and I just can't sort all of that at the moment.” [Interview 12] |
| **Technical-related facilitators (n=0)^d^** | | | | |

^a^Total number of excerpts including multiple mentions from the same persons.

^b^Factors related to the technical devices or processes that made it difficult for the participants to take part in IBI.

^c^IBI: internet-based intervention.

^d^Factors related to the technical devices or processes that made it easier for the participants to take part in IBI.
