# Supplementary material for: Barriers to and Facilitators of Engaging With and Adhering to Guided Internet-Based Interventions for Depression Prevention and Reduction of Pain-Related Disability in Green Professions: Mixed Methods Study
Source: JMIR Ment Health. 2022 Nov 9;9(11):e39122. doi: 10.2196/39122 (PMC9685507; doi:10.2196/39122)
Supplement: Multimedia Appendix 4 [file mental_v9i11e39122_app4.docx]

**Multimedia appendix 4.**

**Figure 1.** Significant group differences in perceived barriers and facilitators based on study affiliation in the quantitative follow-up survey (%). IBI: internet-based intervention.

*Note.* Proportion of participants from PROD-A (*n*=16) and PACT-A (*n*=14) agreeing to the identified barriers and facilitators in the quantitative follow-up survey. Only barrier and facilitating factors with significant group differences (at least *P<*.05) are presented.
